# Low hemoglobin-albumin-lymphocyte-platelet score as a risk marker for stroke-associated pneumonia in acute ischemic stroke: a retrospective cohort study

**DOI:** 10.3389/fneur.2026.1866716

**Published:** 2026-08-13

**Authors:** Zhaoping Wu, Qiruo Sun, Jianmei Zhan, Ming Yang, Tingting Duan, Hong Pan, Jiameng Xu

**Affiliations:** 1 Department of Neurology, Quzhou People’s Hospital, The Quzhou Affiliated Hospital of Wenzhou Medical University, Quzhou, China; 2 Department of Ophthalmology, Quzhou People’s Hospital, The Quzhou Affiliated Hospital of Wenzhou Medical University, Quzhou, China; 3 Department of Neurology, The Affiliated Changsha Central Hospital, Hengyang Medical School, University of South China, Changsha, China

**Keywords:** acute ischemic stroke, HALP score, non-linear association, nutritional status, stroke-associated pneumonia, threshold effect

## Abstract

**Background:**

Stroke-associated pneumonia (SAP) is a common and serious complication of acute ischemic stroke (AIS). The hemoglobin, albumin, lymphocyte, and platelet (HALP) score reflects nutritional, inflammatory, and immune status, but its association with SAP remains unclear.

**Methods:**

We retrospectively analyzed 1,766 consecutive AIS patients admitted between January 2019 and December 2023. HALP was calculated from blood tests obtained within 48 h of admission. Multivariable logistic regression, receiver operating characteristic analysis, subgroup analysis, generalized additive modeling, and two-piecewise logistic regression were used to evaluate the association between HALP and SAP and to explore threshold effects.

**Results:**

SAP occurred in 376 patients (21.3%). HALP was significantly lower in patients with SAP than in those without SAP [35.08 (18.59–51.86) vs. 44.55 (31.29–63.25), *p* < 0.001]. After full adjustment, each 1-unit increase in log2-HALP was associated with a 29% lower SAP risk (OR = 0.71, 95% CI: 0.62–0.83, *p* < 0.001). The highest HALP quartile was associated with a lower SAP risk than the lowest quartile (OR = 0.62, 95% CI: 0.41–0.95; P for trend = 0.018). A non-linear association was identified, with an exploratory threshold at HALP approximately 27.7. Below this threshold, each log2-unit increase in HALP was associated with a 53% lower SAP risk (OR = 0.47, 95% CI: 0.35–0.63, *p* < 0.001), whereas no significant association was observed above it. Adding HALP to the A2DS2 score produced a statistically significant but modest improvement in discrimination (AUC: 0.758 vs. 0.779, *p* = 0.004).

**Conclusion:**

Low HALP score was independently and non-linearly associated with SAP in AIS, with an exploratory threshold near 27.7 that may help inform future risk-stratification research.

## 1 Introduction

Stroke remains one of the leading causes of mortality and long-term disability worldwide, with acute ischemic stroke (AIS) accounting for approximately 80% of all cases (1). Despite substantial advances in reperfusion therapy and acute stroke management, infectious complications during hospitalization continue to pose significant challenges to clinical outcomes (2, 3). Stroke-associated pneumonia (SAP) is the most common and clinically consequential infectious complication, occurring in 10–30% of AIS patients and independently associated with prolonged hospital stays, increased healthcare costs, worse functional outcomes, and higher in-hospital mortality (2, 4, 5).

The pathogenesis of SAP is multifactorial, involving stroke-induced immunodepression syndrome (SIDS), aspiration secondary to dysphagia, impaired cough reflex, and systemic inflammatory dysregulation (2, 6, 7). Neuroendocrine responses to acute brain injury, including activation of the hypothalamic–pituitary–adrenal axis and the sympathetic nervous system, drive lymphocyte apoptosis and suppress adaptive immunity, thereby increasing susceptibility to pulmonary infections (8). Concurrently, malnutrition and hypoalbuminemia—highly prevalent in AIS patients—impair mucosal barrier integrity and innate immune function, further compounding infection risk (9, 10).

Several clinical scoring systems, such as the A2DS2 score (Age, Atrial fibrillation, Dysphagia, Stroke Severity, and Sex), have been developed to predict SAP (11). While these tools incorporate clinical parameters, they do not capture the dynamic nutritional and immunological dimensions of the post-stroke state. Inflammatory biomarkers including C-reactive protein (CRP), neutrophil-to-lymphocyte ratio (NLR), and prognostic nutritional index (PNI) have been evaluated as adjuncts for SAP risk stratification, with variable predictive performance (12–14).

The HALP score, calculated as hemoglobin (g/L) × albumin (g/L) × lymphocyte count (×10^9^/L)/platelet count (×10^9^/L), is a recently introduced composite index that simultaneously reflects nutritional reserve (hemoglobin and albumin), immune competence (lymphocyte count), and inflammatory status (platelet-mediated inflammation) (15). HALP has demonstrated prognostic value in diverse conditions including cancer, sepsis, and cardiovascular disease (15–19). Low HALP scores reflect the triad of malnutrition, immunosuppression, and pro-inflammatory activation that characterizes post-stroke vulnerability to pneumonia. However, to our knowledge, no study has systematically investigated the association between HALP score and SAP in AIS patients, nor has any prior study examined whether this association follows a linear or threshold-dependent dose–response pattern.

In the present retrospective cohort study, we aimed to: (1) evaluate the independent association between HALP score and SAP development in AIS patients; (2) determine whether a non-linear, threshold-dependent relationship exists using generalized additive modeling (GAM) and two-piecewise logistic regression; and (3) assess the incremental predictive value of HALP when combined with established indicators such as CRP and the A2DS2 score.

## 2 Materials and methods

### 2.1 Study design and participants

This study utilized data from a retrospective cohort previously established to investigate complications following ischemic stroke. Patient data were collected at Quzhou People’s Hospital between January 2019 and December 2023. Patients were consecutively enrolled and classified into two groups based on the development of SAP during hospitalization: the SAP group and the non-SAP group. No case–control matching was performed; group differences were addressed using multivariable models. This study was approved by the local Ethics Committee of Quzhou People’s Hospital (Approval Number: 2023–151), which granted a waiver of informed consent owing to the retrospective nature of the study and the use of de-identified data.

**Figure 1:**
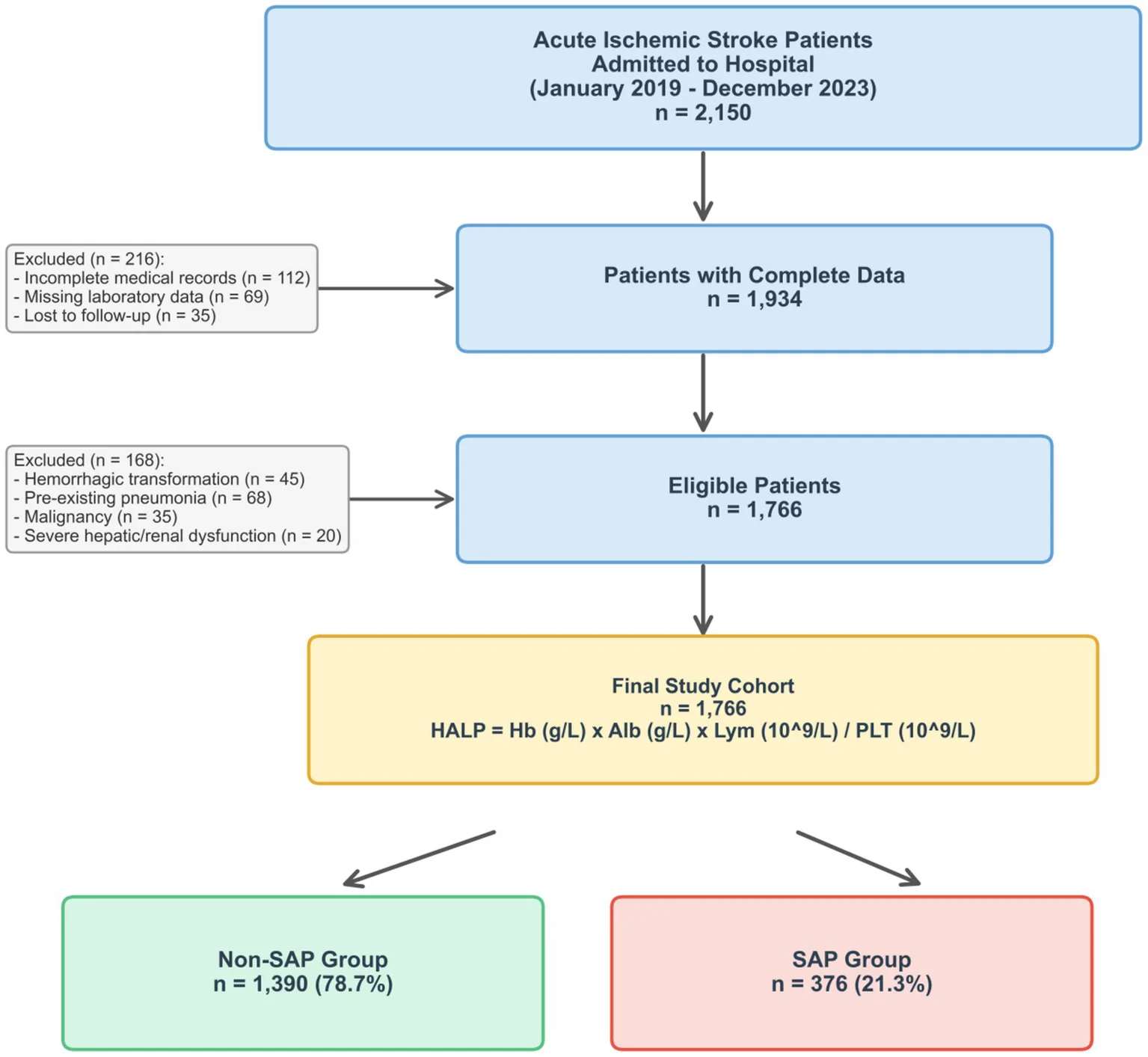
Patient selection flowchart. A total of 2,150 patients admitted with acute ischemic stroke between January 2019 and December 2023 were screened for eligibility. After exclusion of 216 patients with incomplete medical records, missing laboratory data, or lost to follow-up, 1,934 patients with complete data were identified. A further 168 patients were excluded due to hemorrhagic transformation (*n* = 45), pre-existing pneumonia (*n* = 68), malignancy (*n* = 35), or severe hepatic/renal dysfunction (*n* = 20). Ultimately, 1,766 eligible patients were included and stratified into the SAP group (*n* = 376, 21.3%) and the non-SAP group (*n* = 1,390, 78.7%). The HALP score was calculated as: HALP = hemoglobin (g/L) × albumin (g/L) × lymphocyte count (×10^9^/L) ÷ platelet count (×10^9^/L). SAP, stroke-associated pneumonia; HALP, hemoglobin–albumin–lymphocyte–platelet score.

Inclusion criteria were: (1) confirmed diagnosis of AIS within 24 h of symptom onset; (2) brain magnetic resonance imaging (MRI) with diffusion-weighted imaging performed within 48 h confirming acute infarction; (3) blood samples collected within 48 h of symptom onset; and (4) complete data available for HALP score calculation. Exclusion criteria included: (1) incomplete medical records (*n* = 112); (2) missing laboratory data required for HALP calculation (*n* = 69); (3) loss to follow-up (*n* = 35); (4) hemorrhagic transformation (*n* = 45); (5) pre-existing pneumonia at admission (*n* = 68); (6) active malignancy (*n* = 35); and (7) severe hepatic or renal dysfunction (*n* = 20). After exclusions, 1,766 patients constituted the analytical cohort (Figure 1).

Comprehensive baseline data were systematically extracted from electronic medical records. Demographic information included age, sex, temperature, systolic blood pressure (SBP) and diastolic blood pressure (DBP). Vascular risk factors included hypertension, diabetes mellitus, current smoking, atrial fibrillation, and chronic obstructive pulmonary disease (COPD). Clinical assessments performed at admission included: (1) the National Institutes of Health Stroke Scale (NIHSS) for stroke severity (range, 0–42) (38); (2) dysphagia assessment using the Kubota Water Drinking Test (range, 1–5), with dysphagia defined as a score ≥ 3 (39, 40); and (3) the Glasgow Coma Scale (GCS) for level of consciousness (range, 3–15) (41). The need for nasal feeding and the presence of consciousness disturbance were also documented. In addition, the A2DS2 clinical score was calculated according to published criteria (42). For the purpose of this study, pneumonia events occurring within the first 7 days after admission were classified as SAP; community-acquired or pre-existing pneumonia at admission and late-onset pneumonia after 7 days were not considered SAP. Among the included SAP patients, the median time from admission to pneumonia onset was 2 days (interquartile range: 1–4 days).

### 2.2 HALP score calculation

The HALP score was calculated using the formula: HALP = hemoglobin (g/L) × albumin (g/L) × lymphocyte count (×10^9/L)/platelet count (×10^9/L). All blood samples were collected by trained nurses on the second morning after admission (6:00 a.m.) and processed within 2 h. Blood samples were obtained via venipuncture on the morning following admission (6:00–7:00 a.m.) after overnight fasting to ensure consistency in sampling time. Samples were immediately transported to the central laboratory, stored at 4 °C, and analyzed within 2 h. Laboratory measurements included C-reactive protein (CRP, measured by high-sensitivity immunoturbidimetric assay), albumin (measured by the bromocresol green method), white blood cell count (WBC), neutrophil count, hemoglobin, lymphocyte count, platelet count (PLT), fasting glucose (FPG), glycated hemoglobin (HbA1c), triglyceride (TG), total cholesterol (TC), low-density lipoprotein cholesterol (LDL-C), uric acid (UA), homocysteine (HCY), and creatinine. Estimated glomerular filtration rate (eGFR) was calculated using the Chronic Kidney Disease Epidemiology Collaboration (CKD-EPI) equation (43). Higher HALP scores reflect better nutritional status, greater immune competence, and lower platelet-mediated inflammation. For statistical modeling, HALP was log2-transformed to reduce positive skewness and improve linearity assumptions.

### 2.3 Definition of SAP

Stroke-associated pneumonia (SAP) was defined as pneumonia developing within 7 days after admission, in accordance with the Pneumonia in Stroke Consensus Group (PISCES) recommendations (44). Community-acquired pneumonia present at admission was excluded. The diagnosis was established independently by two attending neurologists who were blinded to HALP values and other laboratory parameters, based on the following criteria: at least one of fever >38 °C, abnormal white blood cell count, or altered mental status in patients ≥ 70 years; plus at least two of purulent sputum, new or worsening cough or dyspnea, auscultatory abnormalities, oxygen desaturation, or increased oxygen requirements. Radiological confirmation required new or progressive infiltrates on chest computed tomography. Disagreements were resolved by consensus. Because only the final adjudicated SAP status was retained in the retrospective database, interobserver kappa could not be calculated retrospectively. To address potential temporal ambiguity between early biomarker measurement and early SAP diagnosis, we performed sensitivity analyses excluding patients diagnosed with SAP within the first 1 and 2 days after admission.

### 2.4 Statistical analysis

Normality of continuous variables was assessed using the Kolmogorov–Smirnov test together with graphical and distributional inspection, including histograms, Q-Q plots, skewness, and kurtosis, because formal normality tests may over detect trivial deviations in large samples. All continuous variables were non-normally distributed and are presented as median [interquartile range (IQR)]. Categorical variables are presented as number (percentage). Between-group comparisons used the Mann–Whitney U test or Kruskal-Wallis test for continuous variables and the chi-square test or Fisher’s exact test for categorical variables, as appropriate when low-frequency cells were present.

Multicollinearity was assessed using variance inflation factors (VIF). Variables were excluded from multivariable models if they were arithmetic components of HALP (hemoglobin, albumin, lymphocyte count, PLT), had severe collinearity (WBC and neutrophil count, VIF > 10), or had moderate collinearity that was clinically redundant [LDL-C with TC (VIF = 6.05), A2DS2 with NIHSS and KWDT (VIF = 7.68)]. Among retained covariates, the maximum VIF was 5.03 (NIHSS), indicating no severe multicollinearity. Multivariable logistic regression was performed to identify independent predictors of SAP. Variables were selected for inclusion in the fully adjusted model based on a prespecified clinical and causal framework, rather than purely data-driven stepwise selection. Specifically, variables were considered for adjustment if they met one of the following criteria: (1) established risk factors for SAP from previous literature, including age, atrial fibrillation, dysphagia (KWDT), stroke severity (NIHSS), level of consciousness (GCS), and nasogastric tube feeding; or (2) variables that differed significantly between the SAP and non-SAP groups in univariate analysis. NIHSS, GCS, KWDT, disturbance of consciousness, and tube feeding were incorporated as baseline confounders reflecting stroke severity and airway protective function, rather than as downstream mediators of the HALP-SAP association. Three logistic regression models were constructed: Model 1 (unadjusted); Model 2 (adjusted for age and gender); Model 3 (fully adjusted for age, gender, smoking, hypertension, diabetes, atrial fibrillation, COPD, SBP, DBP, FPG, HbA1C, TG, TC, UA, HCY, CRP, eGFR, NIHSS, and KWDT). Log2-HALP was analyzed as both a continuous variable and in quartile categories.

To assess non-linear associations, generalized additive models (GAM) with penalized natural cubic splines (n_knots = 5, degree = 3) were fitted, with covariates held at their medians to derive partial effects. A two-piecewise logistic regression model was then fitted across all candidate inflection points within the 5th-90th percentile range of log2-HALP (1% steps), selecting the cut-point with maximum log-likelihood. Bootstrap resampling provided 95% confidence intervals for the inflection point, and the data-driven nature of this threshold was considered when interpreting the results. A log-likelihood ratio test compared the piecewise model against the linear model (chi-square, df = 1).

Receiver operating characteristic (ROC) curves and area under the curve (AUC) were computed. AUC confidence intervals used the DeLong method; pairwise comparisons used the DeLong test. Subgroup analyses were conducted across seven predefined clinical variables, with interaction tested by likelihood ratio test. Sensitivity analyses were performed by excluding patients diagnosed with SAP within the first 1 and 2 days after admission, respectively. Additional robustness checks included Fisher’s exact tests for categorical comparisons with potential low-frequency cells and graphical/distributional assessment of continuous variables. All analyses used R (version 4.2.2) and Python (version 3.10). Two-sided *p* < 0.05 was considered statistically significant.

## 3 Results

### 3.1 Baseline characteristics

Among 1,766 AIS patients included, 376 (21.3%) developed SAP during hospitalization. Compared with the non-SAP group, SAP patients were older (median 77 vs. 69 years, *p* < 0.001) and more frequently had atrial fibrillation (35.1% vs. 11.5%), nasal feeding (38.0% vs. 2.4%), disturbance of consciousness (25.5% vs. 2.0%), and COPD (15.7% vs. 4.0%, all *p* < 0.001). No significant differences were observed in sex distribution, smoking status, hypertension, or diabetes prevalence (Table 1). The patient selection process is illustrated in Figure 1. SAP patients had markedly lower HALP scores [35.08 (18.59–51.86) vs. 44.55 (31.29–63.25), *p* < 0.001] and higher CRP (13.62 vs. 2.00 mg/L, *p* < 0.001). Detailed secondary laboratory and metabolic variables, including HALP components, blood cell indices, lipid-related indices, renal function, uric acid, and homocysteine, are provided in Supplementary Table S1. Clinically, SAP patients had higher NIHSS (5 vs. 2), A2DS2 (4 vs. 2), and lower GCS scores (14 vs. 15, all *p* < 0.001).

**Table 1:** Baseline characteristics of patients with acute ischemic stroke, stratified by stroke-associated pneumonia (SAP) status.

Variables	Overall (*N* = 1766)	Non-SAP (*N* = 1,390)	SAP (*N* = 376)	*p*-value
*N*	1,766	1,390	376	
Age (years)	70.00 (61.00–79.00)	69.00 (60.00–77.00)	77.00 (67.00–83.00)	**<0.001**
Gender, *n* (%)				
Male	1,050 (59.5%)	839 (60.4%)	211 (56.1%)	0.153
Female	716 (40.5%)	551 (39.6%)	165 (43.9%)	
Smoke, *n* (%)	649 (36.7%)	511 (36.8%)	138 (36.7%)	1.000
Hypertension, *n* (%)	1,357 (76.8%)	1,070 (77.0%)	287 (76.3%)	0.845
Diabetes, *n* (%)	628 (35.6%)	497 (35.8%)	131 (34.8%)	0.789
Atrial fibrillation, *n* (%)	292 (16.5%)	160 (11.5%)	132 (35.1%)	**<0.001**
Nasal feeding, *n* (%)	177 (10.0%)	34 (2.4%)	143 (38.0%)	**<0.001**
Disturbance of consciousness, *n* (%)	124 (7.0%)	28 (2.0%)	96 (25.5%)	**<0.001**
COPD, *n* (%)	115 (6.5%)	56 (4.0%)	59 (15.7%)	**<0.001**
Laboratory parameters				
HALP score	42.85 (29.34–61.02)	44.55 (31.29–63.25)	35.08 (18.59–51.86)	**<0.001**
CRP (mg/L)	2.82 (1.15–6.91)	2.00 (1.00–4.26)	13.62 (4.00–37.47)	**<0.001**
Clinical characteristics				
NIHSS	3.00 (1.00–6.00)	2.00 (1.00–5.00)	5.00 (2.00–13.00)	**<0.001**
KWDT	1.00 (1.00–1.00)	1.00 (1.00–1.00)	1.00 (1.00–4.00)	**<0.001**
A2DS2	3.00 (2.00–4.00)	2.00 (2.00–4.00)	4.00 (3.00–7.00)	**<0.001**
GCS	15.00 (14.00–15.00)	15.00 (15.00–15.00)	14.00 (12.00–15.00)	**<0.001**

Data are median (interquartile range) or n (%). Secondary laboratory and metabolic variables are shown in Supplementary Table S1. *P*-values were calculated using the Mann–Whitney U test, chi-square test, or Fisher exact test, as appropriate. Bold *p*-values indicate statistical significance (*p* < 0.05). SAP, stroke-associated pneumonia; HALP, hemoglobin-albumin-lymphocyte-platelet score; OR, odds ratio; CI, confidence interval. Other clinical and laboratory abbreviations follow their definitions in the main text.

### 3.2 Baseline characteristics by HALP quartile

Patient characteristics stratified by HALP quartile are shown in Table 2. SAP incidence decreased monotonically across quartiles from Q1 to Q4 (35.1, 19.3, 17.0, and 13.8%, respectively, *p* < 0.001). Higher HALP quartiles were associated with younger age, higher proportion of male patients, and lower rates of atrial fibrillation, nasal feeding, disturbance of consciousness, and COPD. CRP decreased progressively across increasing HALP quartiles (4.00, 2.80, 2.21, and 2.12 mg/L, *p* < 0.001). Detailed secondary laboratory and metabolic trends across HALP quartiles are provided in Supplementary Table S2. NIHSS, A2DS2, and KWDT scores decreased progressively with increasing HALP quartile (all *p* < 0.001).

**Table 2:** Baseline characteristics of patients stratified by quartiles of HALP score (hemoglobin–albumin–lymphocyte–platelet score).

Variables	Q1 (*N* = 442) HALP: 0.15–29.33	Q2 (*N* = 441) HALP: 29.36–42.85	Q3 (*N* = 441) HALP: 42.85–61.01	Q4 (*N* = 442) HALP: 61.03–586.45	*P*-value
*N*	442	441	441	442	
SAP, *n* (%)	155 (35.1%)	85 (19.3%)	75 (17.0%)	61 (13.8%)	**<0.001**
Age (years)	73.00 (64.00–80.00)	70.00 (63.00–79.00)	69.00 (61.00–78.00)	67.00 (57.00–77.00)	**<0.001**
Gender, *n* (%)					
Male	214 (48.4%)	257 (58.3%)	269 (61.0%)	310 (70.1%)	**<0.001**
Female	228 (51.6%)	184 (41.7%)	172 (39.0%)	132 (29.9%)	
Smoke, *n* (%)	133 (30.1%)	148 (33.6%)	169 (38.3%)	199 (45.0%)	**<0.001**
Hypertension, *n* (%)	321 (72.6%)	346 (78.5%)	351 (79.6%)	339 (76.7%)	0.074
Diabetes, *n* (%)	143 (32.4%)	154 (34.9%)	141 (32.0%)	190 (43.0%)	**0.002**
Atrial fibrillation, *n* (%)	95 (21.5%)	70 (15.9%)	69 (15.6%)	58 (13.1%)	**0.007**
Nasal feeding, *n* (%)	80 (18.1%)	45 (10.2%)	33 (7.5%)	19 (4.3%)	**<0.001**
Disturbance of consciousness, *n* (%)	52 (11.8%)	29 (6.6%)	23 (5.2%)	20 (4.5%)	**<0.001**
COPD, *n* (%)	49 (11.1%)	21 (4.8%)	32 (7.3%)	13 (2.9%)	**<0.001**
Laboratory parameters					
HALP score	20.93 (14.37–25.81)	35.74 (32.32–39.24)	50.71 (46.44–55.27)	78.03 (68.26–94.59)	**<0.001**
CRP (mg/L)	4.00 (1.57–14.26)	2.80 (1.34–6.00)	2.21 (1.00–6.00)	2.12 (1.00–4.99)	**<0.001**
Clinical characteristics					
NIHSS	4.00 (1.00–8.00)	3.00 (1.00–5.00)	2.00 (1.00–5.00)	2.00 (1.00–5.00)	**<0.001**
KWDT	1.00 (1.00–2.00)	1.00 (1.00–1.00)	1.00 (1.00–1.00)	1.00 (1.00–1.00)	**<0.001**
A2DS2	3.00 (2.00–5.00)	3.00 (2.00–4.00)	2.00 (2.00–4.00)	2.00 (2.00–4.00)	**<0.001**
GCS	15.00 (13.00–15.00)	15.00 (14.00–15.00)	15.00 (15.00–15.00)	15.00 (14.00–15.00)	**<0.001**

Data are median (interquartile range) or n (%). Secondary laboratory and metabolic variables are shown in Supplementary Table S2. *P*-values were calculated using the Kruskal-Wallis test, chi-square test, or Fisher exact test, as appropriate. Bold *p*-values indicate statistical significance (*P* < 0.05). SAP, stroke-associated pneumonia; HALP, hemoglobin-albumin-lymphocyte-platelet score; OR, odds ratio; CI, confidence interval. Other clinical and laboratory abbreviations follow their definitions in the main text.

### 3.3 Association between HALP score and SAP

Multivariable logistic regression results are presented in Table 3. When treated as a continuous variable, log₂-HALP was significantly associated with SAP across all three models: unadjusted OR = 0.56 (95% CI, 0.50–0.63); age- and gender-adjusted OR = 0.58 (95% CI, 0.51–0.65); and fully adjusted OR = 0.71 (95% CI, 0.62–0.83, *p* < 0.001). In the fully adjusted quartile analysis, compared with Q1 (SAP rate 35.1%), patients in Q2, Q3, and Q4 had significantly lower odds of SAP [OR = 0.61 (0.41–0.91), 0.59 (0.39–0.89), and 0.62 (0.41–0.95), respectively; P for trend = 0.018]. Notably, the adjusted ORs for Q2, Q3, and Q4 were relatively similar, suggesting a plateau effect rather than a strictly linear dose–response relationship across the entire HALP range. The overall trend appeared to be driven primarily by the difference between the lowest quartile and the upper three quartiles. The association remained consistent and statistically significant across all three models, supporting the robustness of findings.

**Table 3:** Multivariable logistic regression analysis of the association between log_2_-transformed HALP score and stroke-associated pneumonia (SAP).

Exposure	Crude model (Model 1) OR (95% CI)	*P*-value	Partially adjusted model (Model 2) OR (95% CI)	*P*-value	Fully adjusted model (Model 3) OR (95% CI)	*P*-value
log₂-HALP (per 1-unit increase)						
HALP (continuous) *N* = 1,766	**0.56 (0.50–0.63)**	**<0.001**	**0.58 (0.51–0.65)**	**<0.001**	**0.71 (0.62–0.83)**	**<0.001**
HALP quartile						
Q1 (HALP: 0.15–29.33) *N* = 442, SAP = 155 (35.1%)	1.00 (Reference)		1.00 (Reference)		1.00 (Reference)	
Q2 (HALP: 29.36–42.85) *N* = 441, SAP = 85 (19.3%)	**0.44 (0.33–0.60)**	**<0.001**	**0.44 (0.32–0.60)**	**<0.001**	**0.61 (0.41–0.91)**	**0.0145**
Q3 (HALP: 42.85–61.01) *N* = 441, SAP = 75 (17.0%)	**0.38 (0.28–0.52)**	**<0.001**	**0.40 (0.29–0.56)**	**<0.001**	**0.59 (0.39–0.89)**	**0.0109**
Q4 (HALP: 61.03–586.45) *N* = 442, SAP = 61 (13.8%)	**0.30 (0.21–0.41)**	**<0.001**	**0.34 (0.24–0.47)**	**<0.001**	**0.62 (0.41–0.95)**	**0.0291**
P for trend		**<0.001**		**<0.001**		**0.0181**

Model 1: Unadjusted (crude model). Model 2: Adjusted for age and gender. Model 3: Adjusted for age, gender, smoking status, hypertension, diabetes mellitus, atrial fibrillation, COPD, systolic blood pressure, diastolic blood pressure, fasting plasma glucose, HbA1C, triglycerides, total cholesterol, uric acid, homocysteine, C-reactive protein, eGFR, baseline NIHSS score, and KWDT. Variables excluded from Model 3 because of collinearity or overlap with HALP are summarized in Supplementary Table S3. Model 3 was fitted using the final imputed analytic dataset (*N* = 1766). P for trend was calculated by entering the quartile number (1–4) as a continuous ordinal variable in the logistic regression model. Bold OR and *P*-values indicate statistical significance (*P* < 0.05). SAP, stroke-associated pneumonia; HALP, hemoglobin-albumin-lymphocyte-platelet score; OR, odds ratio; CI, confidence interval. Other clinical and laboratory abbreviations follow their definitions in the main text.

### 3.4 Subgroup analysis

**Figure 2:**
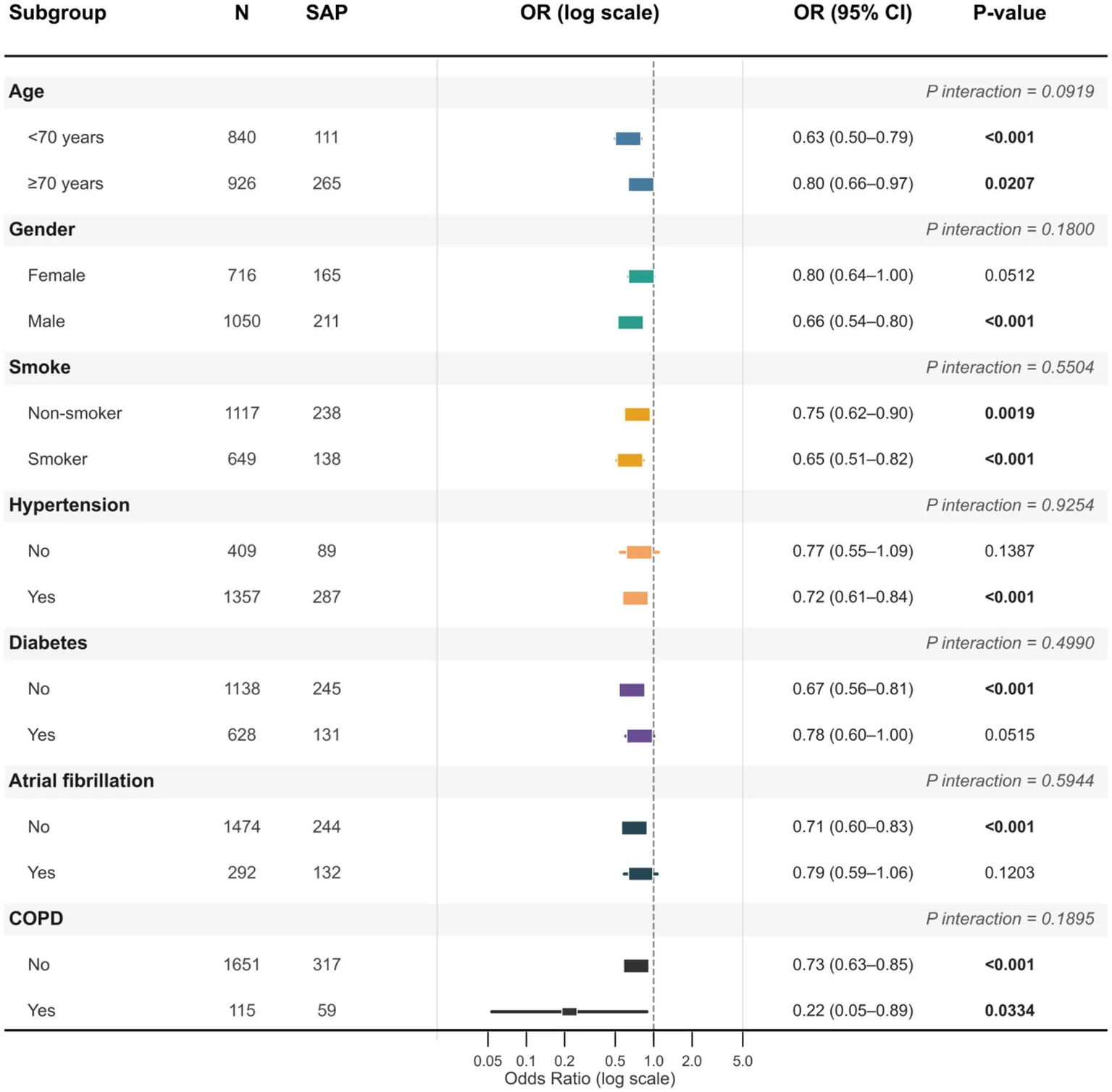
Subgroup analysis of the association between HALP score and stroke-associated pneumonia (SAP). Forest plot showing odds ratios (*ORs*) and 95% confidence intervals (*CIs*) for the association between log2-HALP and SAP across predefined subgroups. Models used the Model 3 adjustment set, excluding the subgroup variable. Square size reflects stratum sample size. Interaction *p*-values were calculated using likelihood ratio tests. In the COPD-Yes stratum, *HbA1C* was additionally excluded because of quasi-complete separation.

Subgroup analyses are presented in Figure 2. The inverse association between HALP score and SAP was consistently observed across all predefined clinical strata, including age groups, sex, smoking status, hypertension, diabetes, atrial fibrillation, and COPD. All *p*-values for interaction were non-significant (all > 0.05), indicating no statistical evidence that the HALP-SAP association differed across these subgroups. Because this was a single-center retrospective study, the apparent consistency across subgroups should be interpreted cautiously and validated in external cohorts.

### 3.5 Diagnostic performance and incremental value

**Figure 3:**
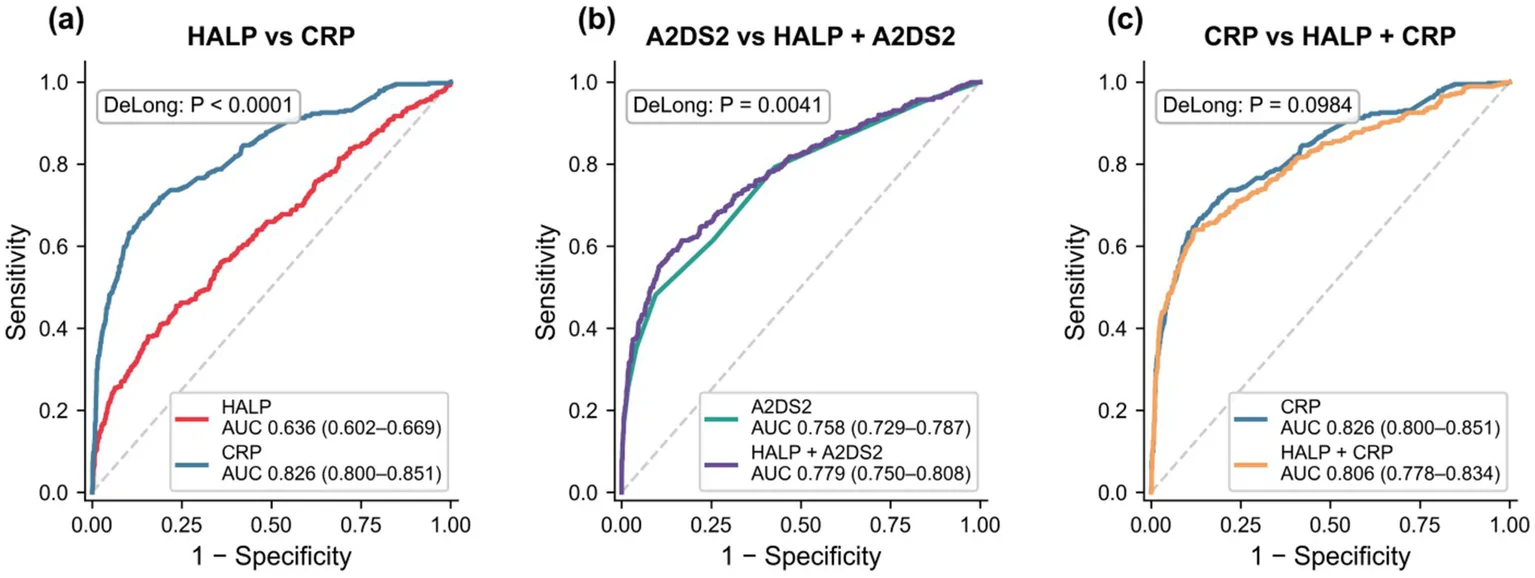
Receiver operating characteristic curves for predicting stroke-associated pneumonia. **(a)** Comparison of HALP with CRP. **(b)** Comparison of A2DS2 with the combined HALP + A2DS2 model. **(c)** Comparison of CRP with the combined HALP + CRP model. AUCs with 95% CIs are reported in the legends, and DeLong test *P* values are displayed in each panel. ROC, receiver operating characteristic; AUC, area under the curve; CI, confidence interval; HALP, hemoglobin-albumin-lymphocyte-platelet score; CRP, C-reactive protein; A2DS2, Age, Atrial Fibrillation, Dysphagia, Stroke Severity, and Sex; SAP, stroke-associated pneumonia.

ROC curve analysis (Figure 3) and the corresponding detailed metrics (Table 4) are presented. HALP alone showed moderate discriminatory ability for SAP (AUC = 0.636), whereas CRP exhibited superior performance (AUC = 0.826, DeLong *p* < 0.0001 vs. HALP). The A2DS2 score yielded an AUC of 0.758. Adding HALP to A2DS2 significantly improved the AUC to 0.779 (DeLong *p* = 0.004), indicating a statistically significant but modest incremental improvement beyond the clinical scoring system alone. In contrast, combining HALP with CRP did not significantly improve over CRP alone (AUC = 0.806, DeLong *p* = 0.098), suggesting mechanistic overlap between these two markers in the acute inflammatory domain. Full comparisons of AUC, sensitivity, specificity, accuracy, and DeLong *p*-values are available in Table 4.

**Table 4:** Comparison of diagnostic performance of HALP score, CRP, A2DS2, and their combination models for predicting stroke-associated pneumonia.

Model	AUC	95% CI (AUC)	Sensitivity (%)	Specificity (%)	Accuracy (%)	DeLong *P*-value
HALP	0.636	0.602–0.669	38.0	84.5	74.6	Reference
CRP	0.826	0.800–0.851	66.8	86.4	82.2	vs HALP: *P* < 0.0001
A2DS2	0.758	0.729–0.787	48.1	90.5	81.5	—
HALP + CRP	0.806	0.778–0.834	63.8	88.1	82.9	vs CRP: *p* = 0.0984
HALP + A2DS2	0.779	0.750–0.808	58.0	86.8	80.7	vs A2DS2: *p* = 0.0041

Performance metrics were calculated in the final imputed analytic dataset (*N* = 1766; SAP = 376). For HALP, reciprocal log2-HALP was used as the ROC predictor so that higher scores indicated higher predicted SAP risk. Combination models used predicted probabilities from binary logistic regression. Sensitivity and specificity used the Youden index. AUC confidence intervals and pairwise comparisons used the DeLong method. AUC, area under the ROC curve; ROC, receiver operating characteristic. SAP, stroke-associated pneumonia; HALP, hemoglobin-albumin-lymphocyte-platelet score; OR, odds ratio; CI, confidence interval. Other clinical and laboratory abbreviations follow their definitions in the main text.

### 3.6 Non-linear association and threshold effect

**Figure 4:**
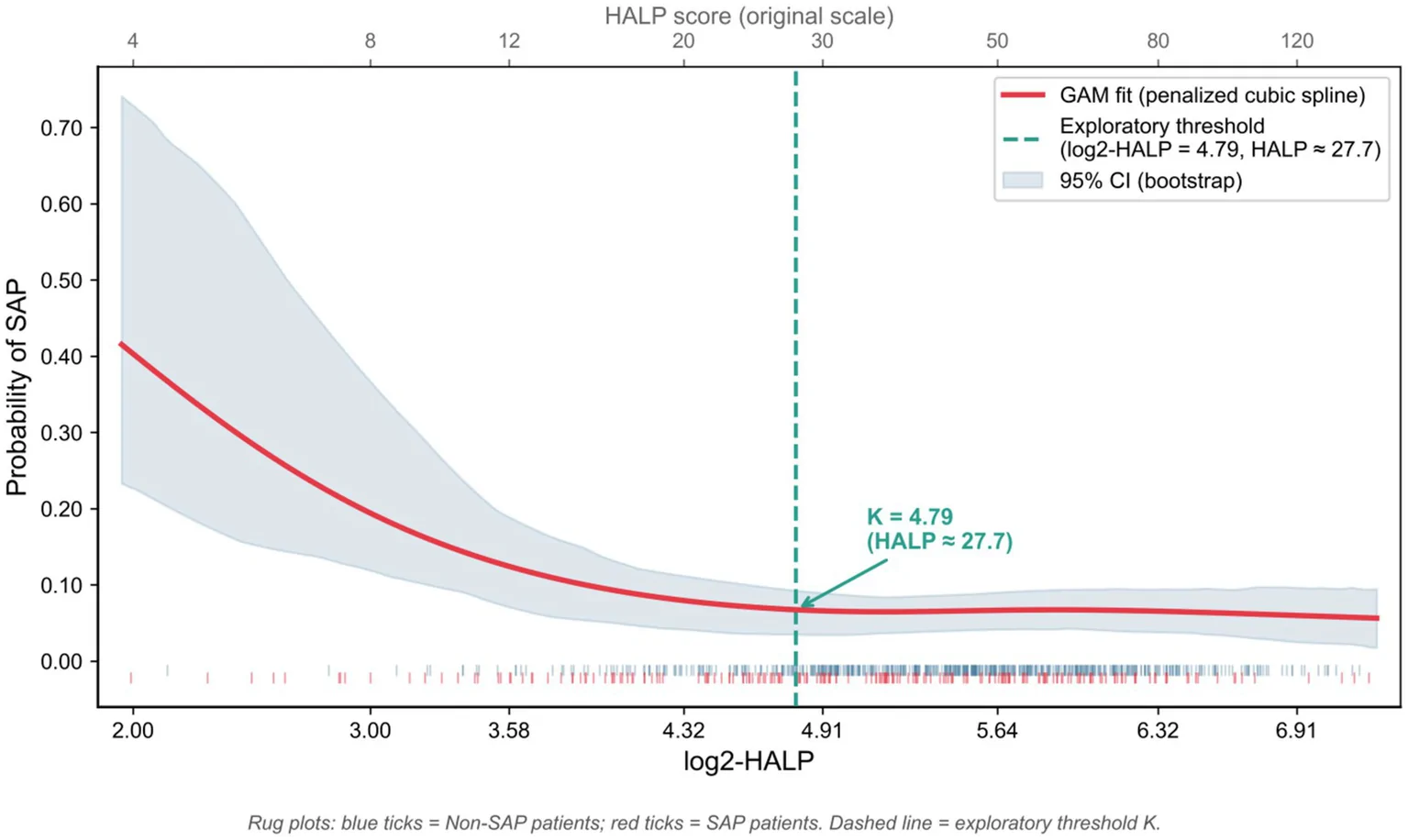
Generalized additive model (GAM) illustrating the non-linear association between log_2_-HALP score and the probability of SAP. The red curve shows the generalized additive model fit, and the shaded area shows the bootstrap 95% confidence interval. The dashed green line marks the inflection point from two-piecewise logistic regression (log_2_-HALP = 4.79; HALP approximately 27.7). The bottom and top x-axes show log_2_-transformed and original HALP values, respectively. Rug plots show individual observations by SAP status.

The inverse association between log₂-HALP and SAP varied across the HALP range. In the linear model, higher log_2_-HALP was associated with lower odds of SAP (OR = 0.71, 95% CI: 0.62–0.83, *p* < 0.0001) (Table 5). The GAM curve showed a steep decline in predicted SAP probability at lower HALP values, followed by a gradual plateau at higher values, with an apparent turning point near HALP 27.7 (Figure 4).

**Table 5:** Threshold effect and linear association between log_2_-transformed HALP score and stroke-associated pneumonia.

SAP outcome	Adjusted OR (95% CI)	*P*-value
Model I: Linear association (log₂-HALP, per 1-unit increase)		
HALP (Model I)	**0.71 (0.62–0.83)**	**<0.0001**
Model II: Two-piecewise logistic regression		
Inflection point (K) log₂-HALP = 4.793 (HALP = 27.72)	95% CI: 3.644–5.491	–
HALP < 27.72 (below K)	**0.47 (0.35–0.63)**	**<0.0001**
HALP ≥ 27.72 (above K)	1.04 (0.81–1.34)	0.7422
Difference (segment 2–1)	**2.22 (1.52–3.24)**	**<0.0001**
Log-likelihood ratio test	–	**<0.001**

Models evaluated linear and two-piecewise associations between log2-HALP and SAP using the final imputed analytic dataset (*N* = 1,766; SAP = 376). Model I treats log2-HALP as a linear term. Model II uses the data-driven inflection point (*K* = 4.793; HALP = 27.72), with the 95% CI estimated by 200 bootstrap resamples. Both models used the Model 3 adjustment set. Segment ORs are per 1-unit increase in log2-HALP. The difference row tests whether segment slopes differ. The log-likelihood ratio test compares the piecewise and linear models. Bold values indicate statistical significance (*P* < 0.05). –, not applicable. K, inflection point; LR, log-likelihood ratio. SAP, stroke-associated pneumonia; HALP, hemoglobin-albumin-lymphocyte-platelet score; OR, odds ratio; CI, confidence interval. Other clinical and laboratory abbreviations follow their definitions in the main text.

Two-piecewise logistic regression identified a similar threshold pattern (Table 5). The estimated inflection point was log₂-HALP = 4.793, corresponding to HALP = 27.72 (bootstrap 95% CI: 12.5–45.4). Below this threshold, each 1-unit increase in log_2_-HALP was associated with markedly lower odds of SAP (OR = 0.47, 95% CI: 0.35–0.63, *p* < 0.0001). Above the threshold, the association was not statistically significant (OR = 1.04, 95% CI: 0.81–1.34, *p* = 0.742). The two segment slopes differed significantly (OR = 2.22, 95% CI: 1.52–3.24, *p* < 0.0001), and the piecewise model provided a better fit than the linear model (log-likelihood ratio test, *p* < 0.001).

### 3.7 Sensitivity and robustness analyses

Sensitivity analyses excluding early SAP cases yielded similar results. After excluding patients diagnosed with SAP within the first 1 day, the association between log₂-HALP and SAP remained significant (*N* = 1,610; SAP events = 220; adjusted OR = 0.686, 95% CI: 0.583–0.808; *p* < 0.001). Similar results were observed after excluding patients diagnosed within the first 2 days (*N* = 1,552; SAP events = 162; adjusted OR = 0.672, 95% CI: 0.563–0.802; *p* < 0.001) (Supplementary Table S4). Fisher exact tests for categorical comparisons were consistent with chi-square results, and distributional diagnostics supported the use of non-parametric tests for continuous variables (Supplementary Table S5).

## 4 Discussion

In this retrospective cohort of 1,766 AIS patients, low HALP score was independently associated with SAP and showed a non-linear threshold pattern. To our knowledge, this is the first study to investigate the HALP-SAP association in AIS patients and to characterize its non-linear pattern. These findings may inform mechanistic understanding and future risk-stratification research, but they should not be interpreted as evidence of causality or as a basis for clinical intervention without prospective validation.

The biological plausibility of the HALP–SAP association is well-grounded in the pathophysiology of post-stroke immunosuppression. Each component of the HALP score contributes a distinct mechanistic dimension. Hemoglobin reflects tissue oxygen delivery capacity; anemia impairs neutrophil and macrophage oxidative burst activity and reduces pulmonary mucociliary clearance, thereby increasing susceptibility to respiratory pathogens (20–22). Albumin is a major acute-phase negative reactant and an indicator of nutritional reserves and hepatic synthetic function; hypoalbuminemia compromises mucosal barrier integrity, reduces opsonization capacity, and has been independently associated with infectious complications in stroke patients (9, 23). Lymphocyte count is the cellular hallmark of adaptive immune competence; post-stroke lymphopenia—a defining feature of SIDS—compromises pathogen clearance and has been directly linked to SAP risk (24). Finally, platelet count serves as an inverse surrogate: thrombocytosis reflects systemic pro-inflammatory activation and platelet-leukocyte interactions that promote pulmonary endothelial injury (25). By integrating these four dimensions, the HALP score provides a single easily obtainable composite measure of the nutritional-inflammatory-immune balance that is mechanistically linked to SAP susceptibility. Beyond these hematological and nutritional parameters, the broader framework of stroke-induced immunosuppression (SIDS) involves complex neuroendocrine and immune pathways. Following acute cerebral ischemia, sympathetic nervous system (SNS) activation and hypothalamic–pituitary–adrenal (HPA) axis stimulation lead to increased circulating catecholamines and cortisol levels, which in turn suppress lymphocyte proliferation and promote a shift from Th1 to Th2 immune responses, favoring anti-inflammatory and pro-infectious states (26, 27). This neuroendocrine-immune crosstalk results in functional impairment of circulating monocytes and reduced antigen-presenting capacity, further compromising host defense against invading pathogens (28, 29). These systemic immunosuppressive changes act in concert with the nutritional-immune deficits captured by HALP to create a permissive environment for SAP development.

In addition to systemic immunosuppression, local host defenses are critically compromised in AIS patients. Dysphagia, present in a substantial proportion of stroke patients, directly increases the risk of aspiration of oropharyngeal contents into the lower respiratory tract. Impaired cough reflex, often coexisting with dysphagia, further diminishes the ability to clear aspirated material, thereby facilitating bacterial colonization and subsequent pneumonia. Moreover, recent evidence suggests that stroke may induce oropharyngeal dysbiosis, characterized by overgrowth of potentially pathogenic respiratory organisms such as *Staphylococcus aureus*, *Pseudomonas aeruginosa*, and enterobacteriaceae, which may translocate to the lower airway through micro-aspiration even in the absence of overt swallowing dysfunction (30, 31). Therefore, the pathogenesis of SAP is multifactorial, arising from the convergence of systemic immunosuppression (SNS/HPA-mediated lymphopenia and monocyte dysfunction), local mechanical deficits (impaired swallowing and cough reflex), and microbial factors (oropharyngeal dysbiosis). HALP, by capturing the pre-existing nutritional-immune reserve before these cascades unfold, may serve as a useful integrative marker of host vulnerability at the intersection of these pathways.

The threshold pattern identified at HALP approximately 27.7 is an exploratory finding of this study. Below this threshold, each doubling of HALP (1-unit increase in log2-HALP) was associated with a 53% reduction in SAP risk, suggesting that the association between nutritional-immune reserve and SAP is strongest among patients with markedly low HALP. Above the threshold, further increases in HALP were not associated with additional risk reduction, suggesting a plateau effect. This non-linear pattern is further supported by our quartile-based analysis: in the fully adjusted model, the ORs for Q2 (0.61), Q3 (0.59), and Q4 (0.62) were remarkably similar, indicating a plateau effect rather than a progressive dose–response relationship across the upper three quartiles. The overall significant trend (P for trend = 0.018) appeared to be driven primarily by the marked difference between Q1 and the upper quartiles, rather than by a stepwise gradient among Q2–Q4. This pattern is consistent with the possibility that HALP functions mainly as a low-reserve risk marker rather than as a strictly linear risk gradient.

From a pathophysiological perspective, the observed plateau effect may be explained by the nature of HALP as a baseline vulnerability marker rather than a dynamic acute-phase reactant. Once a minimum threshold of nutritional-immune competence is achieved (i.e., HALP > 27.7), the host may possess sufficient physiological reserve to resist post-stroke infections, and further improvements in these baseline parameters do not proportionally translate into additional risk reduction. In other words, HALP primarily distinguishes the “nutritionally-immunologically compromised” from the “adequate” population, rather than reflecting a continuous linear gradient of susceptibility. This interpretation aligns with the finding that CRP—a direct acute-phase responder—outperformed HALP in discriminating established SAP, as CRP dynamically captures the ongoing inflammatory response to infection itself, whereas HALP captures pre-existing host vulnerability.

The incremental predictive value of HALP when combined with A2DS2 (AUC: 0.758–0.779, *p* = 0.004) was statistically significant but modest. The A2DS2 score captures stroke severity and clinical risk factors but does not incorporate any biomarker reflecting nutritional or immune status. Our results suggest that integrating HALP with A2DS2 may provide complementary information for early SAP risk stratification. However, such applications remain exploratory and require prospective validation, calibration assessment, and clinical utility analysis before implementation. This complementary relationship contrasts with the finding that HALP + CRP did not significantly exceed CRP alone, reflecting the mechanistic overlap between these two markers in the acute inflammatory response domain.

Comparing our findings with the broader literature on HALP, several parallels emerge. In malignancy settings, low HALP predicts poor prognosis across multiple tumor types (32, 33), with threshold values varying by disease context. In sepsis, HALP has demonstrated predictive value for disease severity and mortality comparable to established scores (18, 34). Our study extends this evidence to the neurological setting and identifies an exploratory HALP threshold that may serve as a potential risk stratification marker. However, as our study was observational and did not evaluate whether interventions targeting HALP modification reduce SAP risk, we emphasize that this threshold should be interpreted as a hypothesis-generating finding rather than a clinical action threshold. Future prospective interventional studies are needed to determine whether nutritional support or immunomodulatory strategies guided by HALP can effectively reduce SAP incidence.

Several inflammatory composite indices have been investigated for SAP prediction in stroke patients. Consistent with previous reports, elevated NLR, PLR, and SII have been associated with increased SAP risk (13, 35–37). However, these markers are primarily derived from white blood cell and platelet counts and predominantly reflect systemic inflammatory activation (45, 46), without directly capturing the nutritional and hematopoietic dimensions of host vulnerability. The Prognostic Nutritional Index (PNI), calculated from serum albumin and lymphocyte count, partially addresses the nutritional-immune interface but does not account for hemoglobin levels, which may reflect baseline physiological reserve and oxygen-carrying capacity. In contrast, the HALP score integrates hemoglobin, albumin, lymphocyte, and platelet counts, thereby offering a more comprehensive assessment of nutritional status, immune competence, and thrombotic-inflammatory balance. The moderate discriminative ability of HALP alone (AUC 0.636) reflects its nature as a baseline vulnerability marker rather than a real-time infection indicator; CRP, which rises acutely with infection onset, predictably performs better as a discriminator of established SAP. The clinical value of HALP lies not in replacing CRP but in providing complementary early risk information available at admission. Nevertheless, our exclusion of patients with severe comorbidities (e.g., malignancies, severe hepatic or renal insufficiency) may limit the generalizability of our findings to more complex stroke populations, and further validation in broader, more representative cohorts is warranted.

Several limitations warrant acknowledgment. First, the retrospective single-center design may limit generalizability, and selection bias cannot be entirely excluded despite multivariable adjustment. Second, HALP was measured as a static single time-point at admission; serial monitoring might better capture dynamic changes in nutritional-immune status. Third, despite comprehensive covariate adjustment, residual confounding by unmeasured factors (e.g., aspiration risk, infarct location, post-stroke immunosuppressive medications) cannot be fully excluded. Fourth, the bootstrap confidence interval for the inflection point was relatively wide (HALP: 12.5–45.4), reflecting biological variability and the inherent uncertainty of data-driven threshold identification. External validation in independent prospective cohorts is essential before clinical implementation. Fifth, although patients with documented pneumonia at admission were excluded and early-onset sensitivity analyses remained consistent, the possibility of subclinical infection not yet clinically manifest at the time of admission cannot be completely ruled out. Thus, residual confounding due to very early subclinical infection may still exist, and our findings should be interpreted with caution. Sixth, only the final adjudicated SAP diagnosis was retained in the retrospective database; therefore, interobserver agreement could not be recalculated using Cohen’s kappa. Seventh, although our quartile-based analysis and nonlinear modeling suggested a plateau effect among higher HALP values, the exact threshold remains data-driven and exploratory. Finally, we did not assess long-term outcomes or evaluate whether nutritional interventions targeting the HALP threshold improve SAP rates. Moving forward, prospective multicenter studies with serial HALP measurements, external validation, calibration assessment, and time-to-event analyses are needed to validate our findings and clarify the temporal relationship between nutritional-immune status and SAP development. Given the observational nature of our study, we cannot infer causality between HALP and SAP, nor can we conclude that modifying HALP through nutritional or immunomodulatory interventions would reduce SAP risk.

## 5 Conclusion

In this retrospective cohort of AIS patients, low HALP score was independently and non-linearly associated with SAP risk, with an exploratory threshold at HALP approximately 27.7. Below this threshold, the inverse association was steep and statistically significant; above it, risk plateaued. Although HALP alone showed moderate discriminatory ability, its combination with A2DS2 produced a statistically significant but modest improvement in predictive performance, suggesting complementary mechanistic information. As a composite index derived from routine clinical laboratory tests, the HALP score is accessible and low cost, and may help inform early risk stratification research in AIS patients. The threshold identified in this study should be viewed as a hypothesis-generating observation that warrants prospective validation before any clinical recommendations can be made.

## Data Availability

The original contributions presented in the study are included in the article/Supplementary material, further inquiries can be directed to the corresponding author.
