# Supplementary material for: Low hemoglobin-albumin-lymphocyte-platelet score as a risk marker for stroke-associated pneumonia in acute ischemic stroke: a retrospective cohort study
Source: Front Neurol. 2026 Aug 13;17:1866716. doi: 10.3389/fneur.2026.1866716 (PMC13518520; doi:10.3389/fneur.2026.1866716)
Supplement: Supplementary file 1 [file Table_1.docx]

**Supplementary Table S1. Variance inflation factors (VIF) for covariates considered in the multivariable logistic regression model**

| **No.** | **Variable** | **VIF** | **Interpretation** | **Remark** |
| --- | --- | --- | --- | --- |
| 1 | Age (years) | 1.906 | No collinearity | *Retained in Model 3* |
| 2 | Gender | 2.439 | Mild collinearity | *Retained in Model 3* |
| 3 | Smoke | 1.735 | No collinearity | *Retained in Model 3* |
| 4 | Hypertension | 1.149 | No collinearity | *Retained in Model 3* |
| 5 | Diabetes | 1.946 | No collinearity | *Retained in Model 3* |
| 6 | Atrial fibrillation | 1.573 | No collinearity | *Retained in Model 3* |
| 7 | COPD | 1.131 | No collinearity | *Retained in Model 3* |
| 8 | SBP (mmHg) | 1.546 | No collinearity | *Retained in Model 3* |
| 9 | DBP (mmHg) | 1.662 | No collinearity | *Retained in Model 3* |
| 10 | FPG (mg/dL) | 2.694 | Mild collinearity | *Retained in Model 3* |
| 11 | HbA1C (%) | 3.409 | Mild collinearity | *Retained in Model 3* |
| 12 | TG (mg/dL) | 1.270 | No collinearity | *Retained in Model 3* |
| 13 | TC (mmol/L) | **6.392** | Moderate collinearity | *Retained in Model 3* |
| 14 | **LDL-C (mmol/L)** | **6.050** | Moderate collinearity | *Excluded: moderate collinearity with TC (VIF > 5)* |
| 15 | UA (μmol/L) | 1.368 | No collinearity | *Retained in Model 3* |
| 16 | HCY (μmol/L) | 1.129 | No collinearity | *Retained in Model 3* |
| 17 | CRP (mg/L) | 1.237 | No collinearity | *Retained in Model 3* |
| 18 | **WBC (×10⁹/L)** | **31.972** | Severe collinearity | Excluded: severe collinearity with neutrophil count (VIF > 10) |
| 19 | **Neutrophil (×10⁹/L)** | **29.673** | Severe collinearity | *Excluded: severe collinearity with WBC (VIF > 10)* |
| 20 | **Lymphocyte (×10⁹/L)** | 4.036 | Mild collinearity | *Excluded: arithmetic component of HALP* |
| 21 | **Hemoglobin (g/L)** | 1.424 | No collinearity | *Excluded: arithmetic component of HALP* |
| 22 | **Albumin (g/L)** | 1.202 | No collinearity | *Excluded: arithmetic component of HALP* |
| 23 | **PLT (×10⁹/L)** | 1.214 | No collinearity | *Excluded: arithmetic component of HALP* |
| 24 | eGFR (mL/min/1.73 m²) | 1.474 | No collinearity | *Retained in Model 3* |
| 25 | NIHSS | **5.031** | Moderate collinearity | *Retained in Model 3* |
| 26 | KWDT | 2.839 | Mild collinearity | *Retained in Model 3* |
| 27 | **A2DS2** | **7.676** | Moderate collinearity | *Excluded: moderate collinearity with NIHSS/KWDT (VIF > 5)* |
| 28 | GCS | 2.589 | Mild collinearity | *Retained in Model 3* |

*VIFs were calculated using the final imputed analytic dataset (N = 1766). SAP (outcome) and HALP score (primary exposure) were excluded from the VIF analysis.*

*VIF < 2: no collinearity; 2 ≤ VIF < 5: mild collinearity (acceptable); 5 ≤ VIF < 10: moderate collinearity (caution); VIF ≥ 10: severe collinearity (problematic, excluded from final model).*

*Variables with VIF >= 10 (WBC and neutrophil count) were excluded from Model 3 due to severe collinearity. The four arithmetic components of HALP (HBG, ALBUMIN, LTC, PLT) were excluded to avoid collinearity with the primary exposure and potential mediation bias. LDL-C was excluded due to moderate collinearity with TC (VIF = 6.050). A2DS2 was excluded due to moderate collinearity with NIHSS and KWDT (VIF = 7.676). TC (VIF = 6.392) and NIHSS (VIF = 5.031) were retained as clinically important covariates; no retained covariate showed severe multicollinearity.*

*Abbreviations: HALP, hemoglobin–albumin–lymphocyte–platelet score; SAP, stroke-associated pneumonia; HBG, hemoglobin; PLT, platelet count; SBP, systolic blood pressure; DBP, diastolic blood pressure; FPG, fasting plasma glucose; HbA1C, glycated hemoglobin; TG, triglycerides; TC, total cholesterol; LDL-C, low-density lipoprotein cholesterol; UA, uric acid; HCY, homocysteine; CRP, C-reactive protein; WBC, white blood cell count; eGFR, estimated glomerular filtration rate; NIHSS, National Institutes of Health Stroke Scale; KWDT, Kubota Water Drinking Test; A2DS2, age, atrial fibrillation, dysphagia, sex, stroke severity; GCS, Glasgow Coma Scale.*

*Bold variable names indicate covariates excluded from the final multivariable model.*
