# Supplementary material for: Low hemoglobin-albumin-lymphocyte-platelet score as a risk marker for stroke-associated pneumonia in acute ischemic stroke: a retrospective cohort study
Source: Front Neurol. 2026 Aug 13;17:1866716. doi: 10.3389/fneur.2026.1866716 (PMC13518520; doi:10.3389/fneur.2026.1866716)
Supplement: Supplementary file 2 [file Table_2.docx]

**Supplementary Table S2. Sensitivity analysis excluding early-onset stroke-associated pneumonia cases**

| **Analysis** | **N** | **SAP events** | **Non-SAP** | **Adjusted OR (95% CI)** | **P-value** |
| --- | --- | --- | --- | --- | --- |
| Primary full cohort | 1766 | 376 | 1390 | 0.715 (0.619-0.826) | <0.001 |
| Excluding SAP onset <=1 day | 1610 | 220 | 1390 | 0.686 (0.583-0.808) | <0.001 |
| Excluding SAP onset <=2 days | 1552 | 162 | 1390 | 0.672 (0.563-0.802) | <0.001 |

*The exposure was log2-transformed HALP score per 1-unit increase. All models used the same covariate set as the fully adjusted Model 3. Early-onset SAP sensitivity analyses excluded patients with SAP onset within 24 h or 48 h after admission, respectively; non-SAP patients were retained as the reference group.*

*Abbreviations: HALP, hemoglobin-albumin-lymphocyte-platelet score; SAP, stroke-associated pneumonia; OR, odds ratio; CI, confidence interval.*
