# Supplementary material for: Low hemoglobin-albumin-lymphocyte-platelet score as a risk marker for stroke-associated pneumonia in acute ischemic stroke: a retrospective cohort study
Source: Front Neurol. 2026 Aug 13;17:1866716. doi: 10.3389/fneur.2026.1866716 (PMC13518520; doi:10.3389/fneur.2026.1866716)
Supplement: Supplementary file 3 [file Table_3.docx]

**Supplementary Table S3. Statistical robustness checks for categorical tests and distributional assumptions**

*Panel A. Fisher exact sensitivity check for categorical variables in Table 1.*

| **Variable** | **Minimum expected count** | **Chi-square P-value** | **Fisher exact P-value** |
| --- | --- | --- | --- |
| Gender, male | 152.4 | 0.1371 | 0.1393 |
| Smoke | 138.2 | 0.9828 | 1.0000 |
| Hypertension | 87.1 | 0.7914 | 0.7832 |
| Diabetes | 133.7 | 0.7423 | 0.7617 |
| Atrial fibrillation | 62.2 | <0.001 | <0.001 |
| Nasal feeding | 37.7 | <0.001 | <0.001 |
| Disturbance of consciousness | 26.4 | <0.001 | <0.001 |
| COPD | 24.5 | <0.001 | <0.001 |

*For all variables in Panel A, chi-square testing was acceptable and Fisher exact sensitivity testing did not change the statistical interpretation.*

*Panel B. Expected-count check for categorical variables in Table 2.*

| **Variable** | **Minimum expected count** | **Chi-square P-value** |
| --- | --- | --- |
| SAP | 93.9 | <0.001 |
| Gender, male | 178.8 | <0.001 |
| Smoke | 162.1 | <0.001 |
| Hypertension | 102.1 | 0.0737 |
| Diabetes | 156.8 | 0.0017 |
| Atrial fibrillation | 72.9 | 0.0074 |
| Nasal feeding | 44.2 | <0.001 |
| Disturbance of consciousness | 31.0 | <0.001 |
| COPD | 28.7 | <0.001 |

*All variables in Panel B had minimum expected counts greater than 5; therefore chi-square testing was acceptable.*

*Panel C. Distributional assessment supporting non-parametric summary statistics.*

| **Variable** | **N** | **Median (IQR)** | **Skewness** | **Excess kurtosis** | **Jarque-Bera P-value** |
| --- | --- | --- | --- | --- | --- |
| Age (years) | 1766 | 70.00 (61.00-79.00) | -0.50 | 0.11 | <0.001 |
| SBP (mmHg) | 1766 | 151.00 (138.00-167.75) | 0.15 | -0.14 | 0.0147 |
| DBP (mmHg) | 1766 | 82.00 (73.00-90.00) | 0.40 | 0.67 | <0.001 |
| Temperature (C) | 1766 | 36.50 (36.20-36.80) | -4.77 | 121.97 | <0.001 |
| HALP score | 1766 | 42.85 (29.34-61.02) | 4.34 | 45.73 | <0.001 |
| Hemoglobin (g/L) | 1766 | 135.00 (124.00-146.00) | -0.54 | 1.59 | <0.001 |
| Albumin (g/L) | 1766 | 38.31 (36.08-40.10) | 0.11 | 5.32 | <0.001 |
| Lymphocyte (10^9/L) | 1766 | 1.58 (1.13-2.14) | 3.22 | 27.86 | <0.001 |
| PLT (10^9/L) | 1766 | 189.00 (151.00-233.00) | 0.92 | 2.44 | <0.001 |
| FPG (mg/dL) | 1766 | 101.63 (90.10-128.44) | 3.00 | 16.44 | <0.001 |
| HbA1C (%) | 1766 | 6.00 (5.52-7.10) | 1.81 | 3.80 | <0.001 |
| TG (mg/dL) | 1766 | 112.93 (79.93-155.00) | 3.44 | 20.85 | <0.001 |
| TC (mmol/L) | 1766 | 4.28 (3.65-5.01) | 0.58 | 1.02 | <0.001 |
| LDL-C (mmol/L) | 1766 | 2.78 (2.18-3.41) | 0.66 | 1.28 | <0.001 |
| UA (umol/L) | 1766 | 311.95 (251.00-380.00) | 0.59 | 0.66 | <0.001 |
| HCY (umol/L) | 1766 | 14.93 (11.71-19.20) | 4.57 | 34.56 | <0.001 |
| CRP (mg/L) | 1766 | 2.82 (1.15-6.91) | 7.54 | 83.53 | <0.001 |
| WBC (10^9/L) | 1766 | 6.97 (5.60-8.68) | 1.85 | 6.70 | <0.001 |
| Neutrophil (10^9/L) | 1766 | 4.54 (3.49-6.18) | 2.20 | 8.41 | <0.001 |
| eGFR (mL/min/1.73m2) | 1766 | 99.30 (79.67-118.90) | 0.91 | 4.03 | <0.001 |
| NIHSS | 1766 | 3.00 (1.00-6.00) | 2.46 | 8.42 | <0.001 |
| KWDT | 1766 | 1.00 (1.00-1.00) | 2.42 | 4.53 | <0.001 |
| A2DS2 | 1766 | 3.00 (2.00-4.00) | 1.32 | 1.58 | <0.001 |
| GCS | 1766 | 15.00 (14.00-15.00) | -3.26 | 12.73 | <0.001 |

*Distributional checks supported reporting continuous variables as median (interquartile range).*

*Abbreviations: IQR, interquartile range; SAP, stroke-associated pneumonia.*
