# Supplementary material for: Low hemoglobin-albumin-lymphocyte-platelet score as a risk marker for stroke-associated pneumonia in acute ischemic stroke: a retrospective cohort study
Source: Front Neurol. 2026 Aug 13;17:1866716. doi: 10.3389/fneur.2026.1866716 (PMC13518520; doi:10.3389/fneur.2026.1866716)
Supplement: Supplementary file 4 [file Table_4.docx]

**Supplementary Table S4. Sensitivity analysis excluding early-onset stroke-associated pneumonia cases**

Adjusted ORs (95% CIs) are reported for log2-HALP per 1-unit increase. Models used the same covariate set as Model 3; non-SAP patients were retained as the reference group.

| **Analysis** | **N** | **SAP events** | **Non-SAP** | **Adjusted OR (95% CI)** | **P-value** |
| --- | --- | --- | --- | --- | --- |
| Primary full cohort | 1766 | 376 | 1390 | 0.715 (0.619-0.826) | <0.001 |
| Excluding SAP onset <=1 day | 1610 | 220 | 1390 | 0.686 (0.583-0.808) | <0.001 |
| Excluding SAP onset <=2 days | 1552 | 162 | 1390 | 0.672 (0.563-0.802) | <0.001 |

Bold P-values indicate statistical significance (P < 0.05).
